# Genome report: first whole genome assembly of *Python regius* (ball python), a model of extreme physiological and metabolic plasticity

**DOI:** 10.1093/g3journal/jkaf197

**Published:** 2025-09-02

**Authors:** Dakota R Hunt, Holly Allen, Thomas G Martin, Sophia N Feghali, Edward B Chuong, Leslie A Leinwand

**Affiliations:** BioFrontiers Institute, University of Colorado Boulder, Boulder, CO 80303, United States; Department of Biochemistry, University of Colorado Boulder, Boulder, CO 80303, United States; BioFrontiers Institute, University of Colorado Boulder, Boulder, CO 80303, United States; Department of Molecular, Cellular, and Developmental Biology, University of Colorado Boulder, Boulder, CO 80309, United States; BioFrontiers Institute, University of Colorado Boulder, Boulder, CO 80303, United States; Department of Molecular, Cellular, and Developmental Biology, University of Colorado Boulder, Boulder, CO 80309, United States; BioFrontiers Institute, University of Colorado Boulder, Boulder, CO 80303, United States; Department of Molecular, Cellular, and Developmental Biology, University of Colorado Boulder, Boulder, CO 80309, United States; BioFrontiers Institute, University of Colorado Boulder, Boulder, CO 80303, United States; Department of Molecular, Cellular, and Developmental Biology, University of Colorado Boulder, Boulder, CO 80309, United States; BioFrontiers Institute, University of Colorado Boulder, Boulder, CO 80303, United States; Department of Molecular, Cellular, and Developmental Biology, University of Colorado Boulder, Boulder, CO 80309, United States

**Keywords:** *Python regius*, ball python, Pythonidae, genome assembly, snake genome

## Abstract

The study of nontraditional model organisms, particularly those exhibiting extreme phenotypes, offers unique insights into adaptive mechanisms of stress response and survival. Snakes, with their remarkable physiological, metabolic, and morphological adaptations, serve as powerful models for investigating these processes. Burmese pythons (*Python bivittatus*) have been used as a model for studying the plasticity of extreme physiological systems. The low contiguity of the *P. bivittatus* genome and rising challenges in obtaining Burmese pythons for study prompted us to sequence, assemble, and annotate the genome of the closely related ball python (*Python regius*). Using a hybrid sequencing approach, we generated a 1.45-Gb genome assembly with a scaffold N50 greater than 61 Mb and a benchmarking universal single-copy ortholog (BUSCO) score of 98%, representing one of the highest quality genomes to date for a member of the Pythonidae family. This assembly provides a valuable resource for studying snake-specific traits and evolutionary biology. Furthermore, it enables exploration of the molecular mechanisms underlying the remarkable cardiac and muscular adaptations in pythons, such as their ability to rapidly remodel their heart following feeding and resist muscular atrophy during prolonged fasting. These insights have potential applications in human health, particularly in the development of therapies targeting cardiac hypertrophy and muscular atrophy.

## Introduction

The study of nontraditional model organisms displaying extreme phenotypes can facilitate the discovery of adaptive mechanisms of stress response and survival. Such mechanisms, which can be difficult to identify or not present in common model organisms, may provide novel insight into human biology and drive innovative therapeutic approaches to human disease (Stenvinkel et al. 2020). Snakes represent one such model due to their significant metabolic, physiological, and morphological adaptations acquired by evolving in environments that would be considered extreme for mammals (Castoe et al. 2008; Vonk and Richardson 2008). Pythons, in particular, have been studied as a vertebrate model of extreme metabolic and organ structural plasticity due to their consumption of large prey after months of fasting (Secor and Diamond 1995, 1998). The postprandial metabolic and physiological remodeling of digesting pythons is unparalleled in known biology in both magnitude and rapidity (Tan et al. 2023; Martin et al. 2024). Pythons display significant organ remodeling, metabolic alterations, and associated changes in gene expression and chromatin remodeling in response to feeding (Secor and Diamond 1995; Castoe et al. 2008; Riquelme et al. 2011; Crocini et al. 2024; Martin et al. 2024). For example, python hearts undergo up to a 40% increase in mass 2–3 d after consuming a large meal, followed by regression to just above their original size within the following days (Secor and Diamond 1995, 1998; Andersen et al. 2005; Riquelme et al. 2011). Intriguingly, pythons do not exhibit skeletal muscle wasting during extended fasting periods (McCue 2007), nor do their hearts undergo atrophy beyond a baseline (Martin et al. 2024).

**Fig. 1.**
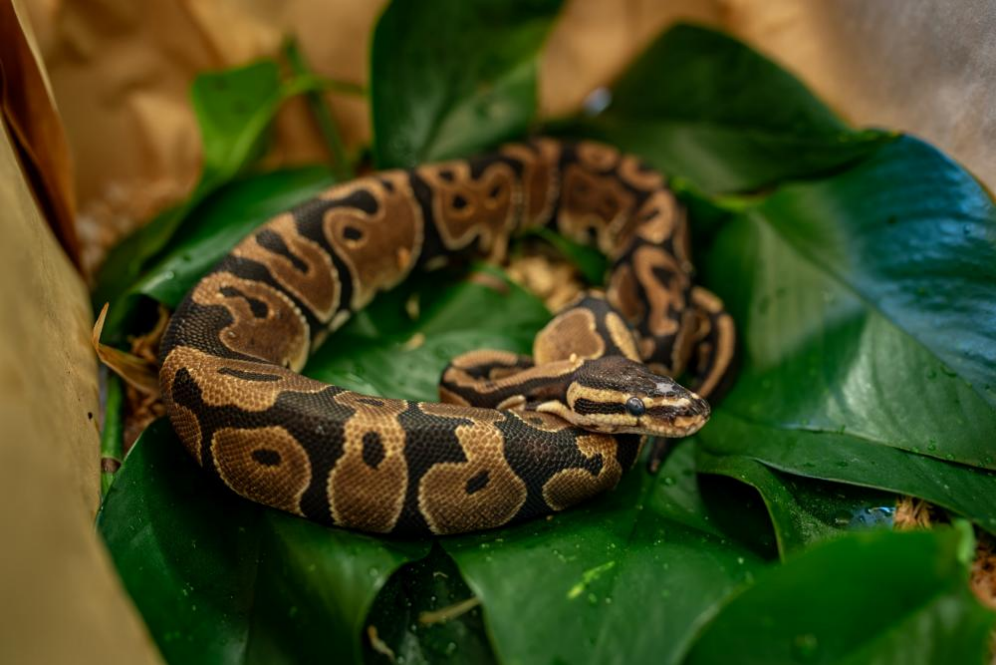
Representative image of *P. regius.* Photo taken by Yuxiao Tan.

Currently, 6 members of the Pythonidae family (taxon ID: 34984) have genome assemblies published at National Center for Biotechnology Information (NCBI). The Burmese python (*Python bivittatus*) was the first member of the Pythonidae family to have its genome sequenced (Castoe et al. 2013), yet the assembly is highly fragmented. Since then, the advent of long-read sequencing technologies, the reduced cost of short-read sequencing, and improvements in genome assembly tools have resulted in more contiguous and accurate genomes, which allows for enhanced comparative genomics studies, easier detection of structural variants, and improved gene prediction (Whibley et al. 2021). While most studies on python biology have utilized Burmese pythons, recent work has included the use of ball pythons (*Python regius*; Crocini et al. 2024). Furthermore, challenges in sourcing Burmese pythons for study, brought on by their invasive species designation in the United States, has led our laboratory and others to employ ball pythons as a model organism (Fig. 1). Here, we present the first *P. regius* genome assembly, and only the second high-quality genome [vertebrate benchmarking universal single-copy ortholog {BUSCO} scores > 90%, contig N50 > 1 Mb] for a member of the Pythonidae family, following the *Liasis olivaceus* genome assembly (GenBank GCA_030867105.1, unannotated). This resource will not only expand our understanding of snake biology and evolution of snake-specific traits but also aid in future studies of unique physiological adaptations, particularly in the realms of cardiac biology and resistance to muscular atrophy. Such work may directly contribute to novel therapeutic approaches targeting pathological cardiac hypertrophy or muscular atrophy in humans.

## Materials and methods

### Specimen origin and tissue collection

All animal procedures were approved by the University of Colorado Boulder Institutional Care and Use Committee (IACUC). The *P. regius* specimen used in this study was a captive-bred adult female received from Bob Clark Reptiles (Oklahoma City, OK, USA). The specimen was housed at the University of Colorado Boulder BioFrontiers Institute vivarium in a room kept at 28 °C and 50% relative humidity. The housing room was subjected to cycling 12-h light–dark periods. This specimen was fasted for 28 d before being fed a rat meal equivalent to 25% of its body weight. The specimen was then fasted for 70 d before being anesthetized with isoflurane and subsequently euthanized via rapid decapitation. The specimen was dissected, and tissues (cardiac ventricle, liver, skeletal muscle, small intestine, brain, kidney, cardiac adipose, visceral adipose, ovary) were collected and snap frozen in liquid nitrogen before being stored at −80 °C.

### DNA extraction and sequencing

High molecular weight (HMW) genomic DNA (gDNA) was extracted and purified using the Monarch HMW DNA Extraction Kit for Tissue from New England Biolabs (Ipswich, MA, USA). Fifteen milligrams of frozen small intestine was biopulverized and further homogenized using the provided pestle. Manufacturer's instructions were followed to extract and purify the DNA. Samples were lysed in a thermal mixer at 56 °C, 1400 rpm for 45 min. Eluted HMW gDNA was incubated overnight at room temperature to ensure homogenous dissolution. The quantity and quality of isolated gDNA were verified by NanoDrop, and an Agilent 2200 TapeStation (Agilent Technologies, Santa Clara, CA, USA) was used to verify the gDNA was indeed of a HMW. A second, standard, gDNA extraction was performed to generate additional Nanopore reads. DNA was extracted from ∼50 mg of small intestine using the Quick-DNA Tissue/Insect Kit (Zymo Research), according to the manufacturer's instructions.

Genomic libraries were prepared separately from both gDNA preps with the Native Barcoding Kit 24 V14 kit (Oxford Nanopore Technologies, Oxford) and sequenced on a R10.4.1 PromethION flow cell (Oxford Nanopore Technologies). The HMW gDNA library was sequenced initially, and to gain increased long-read sequencing depth, the standard gDNA library was subsequently sequenced across 3 flow cells. HMW gDNA extracted via the Monarch kit was also sent to the Genomics Shared Resource Core at the University of Colorado Anschutz Medical Campus (Aurora, CO, USA) for short-read whole genome sequencing. Library preparation was performed using the Ovation Ultralow System V2 library preparation kit (Tecan, Männedorf, Switzerland). Whole genome sequencing was performed using the Illumina NovaSeq X platform (Illumina, San Diego, CA, USA) at a requested coverage of 60×.

### Genome assembly and polishing

Dorado v0.8.2 (Oxford Nanopore Technologies, Oxford, United Kingdom) was used to basecall reads from raw Nanopore sequencing data via the super high accuracy model. Dorado was then used to demultiplex reads with the –no-classify and –emit-fastq flags. NanoStat (De Coster et al. 2018) was used to generate read statistics. The demultiplexed reads were combined into a single fastq file for genome assembly. De novo assembly of the ball python genome was conducted using Flye v2.9.5 (Kolmogorov et al. 2019) with an estimated genome size of 1.5 Gb (–genome-size 1.5 g). Medaka v2.0.1 (Oxford Nanopore Technologies, Oxford, United Kingdom) was used to polish the draft Flye assembly using the raw basecalled Nanopore reads and the r1041_e82_400bps_sup_v5.0.0 model. The bbduk.sh script from BBMap v38.05 (Bushnell) was used to trim adapters from the sequenced Illumina short reads with the following parameters: ktrim = r k = 31 mink = 11 hdist = 1 tpe tbo qtrim = r trimq = 10. Trimmed Illumina reads were then aligned to the Medaka-polished genome by BWA-MEM v0.7.15 (Li 2013). samtools v.1.16.1 (Li et al. 2009; Danecek et al. 2021) was used to create a sorted and indexed bam file containing the aligned reads. Low-quality (-q 10) and unmapped (-F 4) reads were filtered out prior to sorting. Pilon v1.24 (Walker et al. 2014) was used to further diploid-aware polish the genome assembly using the aligned Illumina short reads and default parameters.

### Assembly decontamination and filtering with Blobtoolkit

BlobToolKit v4.4.0 (Challis et al. 2020) was used to perform further quality control (QC) and screen for potential contamination within the genome assembly. BlobToolKit integrates genome coverage information, Basic Local Alignment Search Tool (BLAST) results, and BUSCO analysis output to allow for interactive visualization of a dataset and subsequent filtering of an assembly dataset. To generate coverage information, we aligned the Illumina and Nanopore reads back to the Pilon-polished genome. Illumina reads were aligned using BWA-MEM and basecalled, and demultiplexed Nanopore reads were aligned using minimap2 v2.17 (Li 2018). samtools was used to convert the subsequent sam files to bam files and sort and index the bam files. To generate taxonomic information for each contig in the Pilon-polished assembly, we used the BLASTn 2.7.1+ (Altschul et al. 1990; Camacho et al. 2009). Assembly contigs were queried against the core_nt database v1.1 from the NCBI using the following flags: *-outfmt “6 qseqid staxids bitscore std” -max_target_seqs 10 -max_hsps 1 -evalue 1e-25*. DIAMOND v2.1.10 (Buchfink et al. 2021) is a protein and translated sequence aligner that is significantly faster than BLAST. Diamond makedb was used to create a Diamond-formatted database from the curated, nonredundant UniProtKB/Swiss-Prot protein sequence database. Taxonomic information was added using the “prot.accession2taxid.FULL,” “names.dmp,” and “nodes.dmp” files from NCBI. Diamond was then used to blast the query Pilon-polished assembly against the created database with the following flags: *–outfmt 6 qseqid staxids bitscore qseqid sseqid pident length mismatch gapopen qstart qend sstart send evalue bitscore –sensitive –max-target-seqs 1 –evalue 1e-25*. A BlobTools dataset was created using the BlobTools create command and specified taxonomic information for *P. regius*. The add command was then used to add coverage information, BLASTn/DIAMOND hits, and BUSCO output to the dataset. The BlobTools *filter* command was used to remove all contigs less than 3,000 base pairs from the assembly.

### Purge_dups

Purge_dups v1.0 (Guan et al. 2020) was used to remove low-coverage contigs, collapsed repeat contigs, haplotigs, and contig overlaps from the BlobTools-filtered genome assembly. A configuration file specifying the input Nanopore and Illumina data was generated using the pd_config.py script, and the pipeline was run using the run_purge_dups.py script. The high coverage cutoff was then manually changed, resulting in the following coverage cutoffs: low = 5, mid = 21, and high = 100. The BlobTools-filtered assembly was then purged using these manual cutoffs and the -c and -e flags to ensure high coverage contigs were not removed and that only duplications at the end of contigs were removed. The purged assembly and discarded sequences were then extracted.

### Mitochondrial identification and removal of mitochondrial contigs

MitoHiFi v3.2.1 in contigs mode (Uliano-Silva et al. 2023) was then used to query the purged assembly against the existing *P. regius* mtDNA genome (GenBank: AB177878.1; Dong and Kumazawa 2005) and identify mitochondrial contigs. Identified contigs were removed from the assembly using SeqKit v0.9.0 (Shen et al. 2016). In order to produce an annotated mitogenome from our sample, we ran MitoHiFi in reads mode with the demultiplexed Nanopore DNA sequencing reads and the GenBank *P. regius* mtDNA genome as a reference genome.

### Genome scaffolding

ntLink v1.3.11 (Coombe et al. 2021, 2023) was used to scaffold the cleaned contig assembly utilizing the long Nanopore sequencing reads. To maximize scaffolding gains, ntLink_round was used to run 3 iterative rounds of scaffolding using the following parameters: k = 32, w = 250, v = 1, sensitive = True, and rounds = 3. To assess whether any scaffolds may represent complete chromosomes, TelomereSearch.py (McGowan 2021) was used to search for telomeric sequence at the ends of scaffolds with the following parameters: -l 100 -t 0.4 and -l 200 -t 0.4.

### Genome quality control

The Quality Assessment Tool (QUAST) v5.3.0 (Mikheenko et al. 2023) was used to generate genome metrics (total length, N50, L50, etc.) and assess genome quality. BUSCO v5.8.2 (Manni et al. 2021) is a tool used to assess genome assembly quality and completeness by identifying the presence and integrity of a set of universally conserved single-copy orthologous genes across species. *P. regius* assemblies were input into BUSCO and run against the following lineages: “sauropsida_odb10” (7,480 BUSCOs) and “vertebrata_odb10” (3,354 BUSCOs). *P. bivittatus* (NCBI RefSeq assembly GCF_000186305.1) and *Candoia aspera* (NCBI RefSeq assembly GCF_035149785.1) genomes were also analyzed for comparison.

### Repeat modeling and masking

RepeatModeler v2.0.6 (Flynn et al. 2020 ) was used to identify and model repeats in the scaffolded assembly (Supplementary Files 2 and 3). The repeat family library from this de novo identification was merged with repeats identified in the serpentes taxon and in serpentes descendants from the Dfam database v3.8. RepeatClassifier (part of RepeatModeler) was used to classify the repeats in this merged library. RepeatMasker v4.1.7 (Smit et al. 2013) was used to softmask the repeats contained within the merged library, generate repeat statistics, create a General Feature Format (GFF) repeat annotation file, and produce a RepeatMasker output file (Supplementary File 4) with the with the -a, -s, and -xsmall flags.

### RNA extraction and sequencing

Frozen tissues (cardiac ventricle, liver, skeletal muscle, small intestine, brain, kidney, cardiac adipose, visceral adipose, ovary) were homogenized in TRIzol with a mechanical homogenizer (Omni International) and incubated for 5 min at room temperature. Chloroform was added to the tubes at 1:5 vol/vol ratio, and the samples were shaken, before incubating for 15 min at room temperature. Samples were subsequently centrifuged (12,000 Relative Centrifugal Force (RCF), 15 min, 4 °C), and the aqueous upper layer was collected and combined with an equal volume of 75% ethanol and briefly vortexed. The solution was then applied to a RNeasy mini column (Qiagen) to help remove impurities. The manufacturer's protocol was followed to wash the column in subsequent steps, and then the RNA was eluted in RNase-free water. RNA samples were sent to Novogene Corporation (Sacramento, CA) for cDNA library preparation and short-read Illumina NovaSeq sequencing at a minimum 50 million read-pair depth per sample. For long-read Nanopore sequencing, an RNA library was prepared with the PCR cDNA Barcoding kit (SQK-PCB111.24, Oxford Nanopore Technologies) and sequenced on an R9.4.1 PromethION flow cell (Oxford Nanopore Technologies).

### RNA read processing and alignment

Dorado v0.8.2 (Oxford Nanopore Technologies) was used to basecall Nanopore reads with the super high accuracy model and the –no-trim flag. Basecalled reads were then demultiplexed with Dorado and the –emit-fastq –no-trim flags provided. pychopper v2.7.10 (Oxford Nanopore Technologies) was used to polish and trim the cDNA reads. The high-confidence full-length reads and rescued reads from each tissue sample were concatenated and mapped to the assembled genome with minimap2 v2.17 with the splice preset (-x splice). Illumina reads were trimmed with bbduk.sh and the trimming parameters as for the Illumina WGS reads (see above). A HISAT2 index was constructed with HISAT2 v2.1.0 (Kim et al. 2019), and trimmed Illumina reads were aligned to the final genome assembly with sensitive alignment parameters and the –dta flag to generate alignments suitable for transcript assembly. We also utilized previously published raw RNA-seq data from the ball python heart (BioProject PRJNA1142400). This data was trimmed with bbduk.sh and the following parameters: ktrim = r, qtrim = r, trimq = 10, k = 23, mink = 11, hdist = 1, maq = 10, minlen = 25, and literal = AAAAAAAAAAAAAAAAAAAAAAA. Trimmed reads were aligned to the scaffolded assembly using HISAT2 as discussed above. All SAM files containing minimap2 and HISAT2 alignments were converted to BAM files, sorted, and indexed with samtools. The final short-read RNA-seq bam files from all samples (9 new tissue samples, 6 samples from BioProject PRJNA1142400) were merged with the samtools merge function.

### Protein-coding structural gene annotation

To annotate protein-coding genes, we utilized EVidenceModeler (EVM) v2.1.0 to combine ab initio gene predictions, protein alignments, and transcript alignments. First, the BRAKER3 annotation pipeline (Gabriel et al. 2024) was used to generate high-confidence gene predictions. BRAKER3 incorporates RNA-seq and protein data and uses the gene prediction tools GeneMark-ETP (Brůna et al. 2024) and AUGUSTUS (Stanke et al. 2008) to train and predict genes with high support from extrinsic evidence. Final predictions from both GeneMark and AUGUSTUS are combined using TSEBRA (Gabriel et al. 2021) to produce the final BRAKER output. BRAKER3 was run with aligned short-read RNA-seq reads (BAM files) as extrinsic evidence and the prepartitioned vertebrate set of proteins from the OrthoDB v.12 database (Kriventseva et al. 2015). GeMoMa v1.9 (Keilwagen et al. 2018, 2019) was used for homology-based gene prediction. We used the *Xenopeltis unicolor* annotation (Peng et al. 2023) with the following parameters: GeMoMa.Score = ReAlign, AnnotationFinalizer.r = NO, o = true, and tblastn = true. We aligned the *P. bivittatus* (RefSeq assembly GCF_000186305.1) and *C. aspera* (RefSeq assembly GCF_035149785.1) proteomes to the final *P. regius* genome assembly using miniprot v0.14-r265 (Li 2023). As our lab has a special interest in the sarcomeric protein titin, we also aligned all vertebrate titin sequences in the NCBI database. Default parameters were used with the –gff and -I flags included. The final gff3 files from each alignment were concatenated into a single gff3 file. Output files from AUGUSTUS, final TSEBRA/BRAKER3 output, GeMoMa, and miniprot were converted to EVM-compatible gff3 formats using the utility scripts provided with the EVidenceModeler software. EVM was used to combine BRAKER, GeMoMa, and miniprot outputs with default parameters and the following flags: segmentSize = 1000000 and overlapSize = 100000. For each input source, an input type and weight must be provided to EVM. The following input types and weights were used: original AUGUSTUS predictions (ABINITIO_PREDICTION, 1), miniprot alignments (PROTEIN, 5), GeMoMa (OTHER_PREDICTION, 1), BRAKER3 final predictions from AUGUSTUS or GeneMark.hmm3 (OTHER_PREDICTION, 5), and BRAKER3 final predictions from GeneMark S-T (OTHER_PREDICTION, 10). In order to incorporate RNA-seq data, we utilized StringTie v2.2.3 (Pertea et al. 2015; Kovaka et al. 2019; Shumate et al. 2022) in mixed reads processing mode (–mix flag) to assemble a transcriptome. The EVM output gff3 file was used as a reference annotation, with the long-read Nanopore bam file and the combined short-read bam file as inputs. Transcript sequences were extracted using the agat_sp_extract_sequences.pl script from AGAT (Dainat). We then utilized PASA v2.5.3 (Haas et al. 2003, 2008) to clean transcripts and generate transcript alignments with the following parameters: MIN_PERCENT_ALIGNED = 75, MIN_AVG_PER_ID = 95, NUM_BP_PERFECT_SPLICE_BOUNDARY = 0, –ALIGNERS blat, gmap, and minimap2. ORFs were extracted by TransDecoder using the pasa_asmbls_to_training_set.dbi script within PASA. EVM was rerun to incorporate PASA assemblies using the same parameters and weights as before, with the following exceptions: miniprot (PROTEIN, 2), PASA assemblies (TRANSCRIPT, 10), and TransDecoder ORF predictions (OTHER_PREDICTION, 5). Because EVM only produces 1 consensus gene structure and does not account for alternative splicing isoforms, we reran StringTie as before, but with the final EMV gff3 provided as the reference genome annotation file. TransDecoder v5.7.1 (Haas 2025) was used to generate ORF predictions from the final StringTie transcript set. Transcripts were extracted with gtf_genome_to_cdna_fasta.pl, and the StringTie gtf file was converted to gff3 format with gtf_to_alignment_gff3.pl. The LongOrfs function of TransDecoder was used to generate best candidate ORF predictions with the complete_orfs_only flag to remove any incomplete ORFs. TransDecoder.Predict was then run with the –single_best_only flag to ensure only 1 CDS prediction per transcript. A final genome-based gff3 annotation file was generated with cdna_alignment_orf_to_genome_orf.pl. The final EVM gff3 file and final TransDecoder gff3 file were merged with agat_sp_merge_annotations, and the agat_sp_filter_incomplete_gene_coding_models.pl script was run to remove any incomplete gene-coding models.

### Protein-coding functional gene annotation

To functionally annotate protein-coding genes, we used the Eukaryotic Non-Model Transcriptome Annotation Pipeline (EnTAP; Hart et al. 2020). First, we extracted coding sequences (CDSs) from our final gff3 annotation file with agat_sp_extract_sequences.pl. These sequences were used as input to the EnTAP pipeline, which was run with default parameters with frame selection enabled and the NCBI RefSeq vertebrate_other database of protein sequences for similarity searching. EnTAP functionally annotates proteins using similarity searching with DIAMOND (Buchfink et al. 2015, 2021) and orthologous group assignment (protein domains, gene ontology terms, KEGG (Kyoto Encyclopedia of Genes and Genomes) pathways) with eggNOG-mapper (Huerta-Cepas et al. 2019; Cantalapiedra et al. 2021).

### Noncoding RNA annotation

To predict and annotate transfer RNAs (tRNAs) we used tRNAscan-SE v2.0.12 (Chan et al. 2021) with default parameters. To annotate additional noncoding RNAs (ncRNAs), we used Infernal (“INFERence of RNA ALignment”) v1.1.5 (Nawrocki and Eddy 2013). The cmscan program was used with the following parameters: -Z 2896.184112, –cut_ga, –rfam, –nohmmonly, and –fmt 2. The Rfam library of covariance models was used to search the final genome assembly with clan information provided by the Rfam.clanin file. All tRNA and rRNA annotations were removed from the cmscan output. Furthermore, cmscan occasionally assigns 2 ncRNAs at the same location but on opposite strands. In these instances, the ncRNA with the lower cmscan score was manually removed.

### Genome annotation polishing

All transcripts with partial coding frames or no assigned functional annotation by EnTAP were removed from the final annotation with agat_sp_filter_feature_from_kill_list.pl. The protein-coding, tRNAscan, and final cmscan annotations were combined. tRNAs that overlapped with coding sequences were manually removed. Protein product names were assigned to mRNA features and their child CDS features based on the results of the EnTAP annotation. Feature IDs were then cleaned and reassigned using agat_sp_manage_IDs.pl. Protein product names were manually cleaned in some instances to align with NCBI specifications. To remove a handful of remaining overlapping genes, agat_sp_fix_overlaping_genes.pl was used to merge gene features with overlapping CDS features and designate the different mRNAs as isoforms. Accession numbers from EnTAP annotation were converted to gene symbols using the NCBI database. Any genes that were annotated with multiple differing gene symbols were manually inspected and fixed to separate merged genes (2 genes annotated as 1 likely due to transcriptional read-through between the genes, resulting in an annotated mRNA that spanned both genes) using a custom Python script (Supplementary File 5). A custom Python script was used to check for any remaining genes that contained nonoverlapping CDS sequences (Supplementary File 6). Identified genes were manually inspected and split into separate genes if necessary. Finally, a custom Python script was used to remove mRNAs without any untranslated regions (UTRs) that overlapped exactly in their CDS with an mRNA that did contain UTR regions (Supplementary File 7).

### Proteome BUSCO analysis

Proteomes from the final *P. regius* annotation, RefSeq *P. bivittatus* annotation, and RefSeq *C. aspera* annotation were analyzed using BUSCO with the vertebrata_odb10 database. Isoforms were not removed from the proteome datasets, which results in an increased count of duplicate BUSCOs. Choosing the longest isoform only and then conducting BUSCO analysis fixes this problem but also can lead to a decreased overall complete count, as the longest isoform is not always the “best” isoform. The complete scores reflected below are overall complete scores (single and duplicate).

### Comparative genomics analysis

The nonredundant proteomes for *P. regius*, *P. bivittatus*, and *C. aspera* were used for orthologous cluster analysis with OrthoVenn3 (Sun et al. 2023). The OrthoMCL algorithm was used with an E-value of 1e-5 and an inflation value of 1.50.

## Results and discussion

### Assembly

We generated 51.5 Gb of Nanopore ONT reads with a mean length of 3,847.6 bp and a read length N50 of 6,377 bp (see Supplementary Table 1 in Supplementary File 1). The initial de novo Flye assembly using these reads consisted of 3,056 contigs with a reported mean coverage of 28×. Pilon was used to polish the genome assembly with aligned Illumina reads, which had a mean coverage of 56× (see Supplementary Table 2 in Supplementary File 1). We then cleaned the genome by removing all contigs smaller than 3,000 bp, low-coverage contigs, contigs identified as mitochondrial, and contigs designated as haplotigs or collapsed repeats. While no complete mitochondrial contig was present in our assembly, we were able to generate a mitochondrial genome using the raw Nanopore DNA reads and the published *P. regius* mitochondrial genome from GenBank. Our mitogenome was 17,200 bp long, slightly shorter than the 17,245-bp genome from GenBank. It contained all the expected genes with the exception of a tRNA-Trp, which was labeled as a termination tRNA in our assembly. While this mitogenome is not included in our final assembly, it is provided in the Supplementary material (Supplementary File 8). We detected no evidence of contamination from bacteria or other organisms in our assembly. The final set of cleaned contigs was scaffolded using ntLink and the processed Nanopore reads. No scaffolds contained telomeric sequences at both ends, suggesting that none of the scaffolds present in the assembly represent complete chromosomes.

**Fig. 2.**
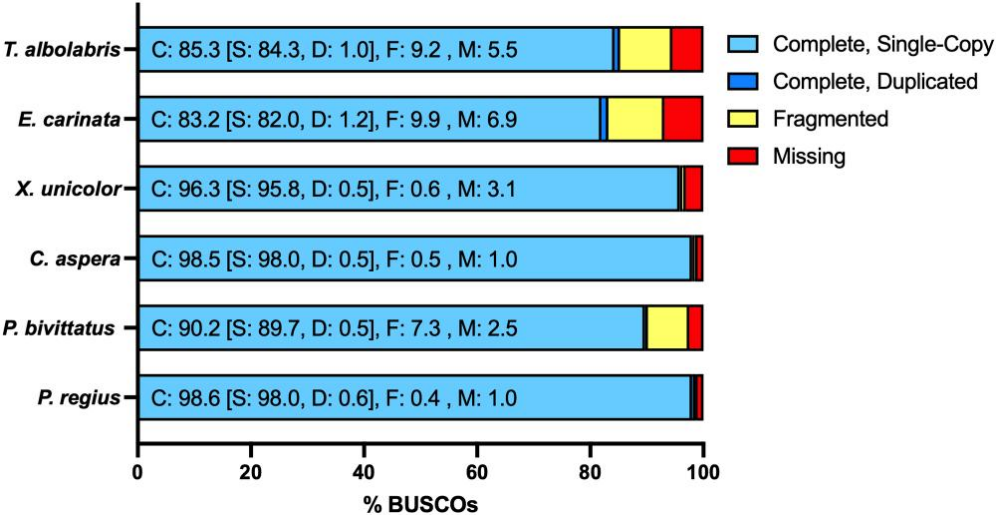
Genome BUSCO scores calculated using the vertebrata_odb10 database. Numbers in the bar plot represent percentages of the database BUSCOs (*n* = 3354) belonging to each category. *Trimeresurus albolabris* (Niu et al. 2024), *Elaphe carinata* (Fan et al. 2023), *Xenopeltis unicolor* (Peng et al. 2023), *C. aspera* (NCBI RefSeq assembly GCF_035149785.1), and *P. bivittatus* (NCBI RefSeq assembly GCF_000186305.1) genome BUSCO scores are provided for comparison.

The final genome assembly was 1.45 Gb (Supplementary File 9), virtually identical in size to the genomes of the closely related Burmese python and reticulated python (*Malayopython reticulatus*), which are both approximately 1.44 Gb (De Smet 1981; Castoe et al. 2013). Unsurprisingly, this assembly was also significantly more contiguous than the Burmese genome (NCBI RefSeq assembly GCF_000186305.1), which was sequenced over a decade ago via Illumina and Roche 454 sequencing. The final scaffolded assembly consisted of 804 scaffolds, approximately 49 times less than the 39,112 scaffolds of the Burmese python assembly (Table 1). The scaffold N50 value was significantly higher for the assembled ball python genome (61.3 Mb) compared to that of the current Burmese python genome (214.0 Kb). The final assembly has a BUSCO score of 98% (vertebrate complete and single-copy orthologs; Table 2; Fig. 2), notably higher than 89.7% for the Burmese python genome. The reduced BUSCO score for the Burmese genome is due to a higher percentage of fragmented and missing orthologs compared to this assembly. Furthermore, the ball python genome has greater completeness compared to several recently assembled snake genomes (Fan et al. 2023; Peng et al. 2023; Niu et al. 2024) and is similar to the high-quality genome assembly of the viper boa (*C. aspera*; Fig. 2). Consequently, this assembly represents one of the most contiguous genomes for a member of the Pythonidae family to date and is on the higher end in terms of BUSCO completeness when compared to recently published snake genomes.

**Table 1.** Quality statistics for the Flye, cleaned contig, and final scaffolded *P. regius* genome assemblies.

	Flye	Cleaned contig assembly	ntLink scaffolded—final *P. regius* assembly	Python_molurus_bivittatus-5.0.2 (GCF_000186305.1)
**Length (bp)**	1,467,955,681	1,449,099,357	1,448,092,056	1,435,052,152
**Contigs**	3,056	1,078	955	274,244
**Largest contig (bp)**	81,416,170	81,396,585	91,593,816	103,905
**Contig N50 (bp)**	18,131,975	18,346,808	27,270,377	10,658
**Contig N90 (bp)**	4,078,938	4,386,424	5,244,159	2,731
**Contig L50**	20	19	14	38,694
**Contig L90**	85	81	65	134,497
**Contig auN**	27,705,154.3	28,045,570.5	35,164,974.6	13,374.7
**GC (%)**	40.29	40.21	40.20	39.61
**Scaffolds**	ND	ND	804	39,112
**Largest scaffold (bp)**	ND	ND	122,704,369	1,452,584
**Scaffold N50 (bp)**	ND	ND	61,335,313	213,970
**Scaffold N90 (bp)**	ND	ND	8,700,500	52,434
**Scaffold L50**	ND	ND	10	1,939
**Scaffold L90**	ND	ND	35	6,957
**Scaffold auN**	ND	ND	53,749,847.8	274,923.4
**# *N*'s per 100** **kbp**	0	0	38.14	3,519.16

The statistics for the latest NCBI RefSeq version of the *P. bivittatus* genome are provided for comparison. N50 and N90 represent the length of the shortest contig or scaffold at which 50% (N50) or 90% (N90) of the total assembly length is reached. L50 and L90 indicate the smallest number of contigs or scaffolds that sum to 50% (L50) or 90% (L90) of the total assembly length. auN is a measure of genome contiguity representing the area under the Nx curve. GC percentage represents the GC content of the genome assembly. # N's per 100 kbp is the average number of uncalled bases (N's) per 100,000 assembly bases. See QUAST documentation for further details.

**Table 2.** Genome BUSCO scores for the Flye, cleaned contig, and final scaffolded *P. regius* assemblies.

	Flye	Cleaned contig assembly	ntLink scaffolded—final *P. regius* assembly	Python_molurus_bivittatus-5.0.2 (GCF_000186305.1)
**Vertebrata**				
**Complete**	98.6%	98.6%	98.6%	90.2%
**Single**	98.0%	98.0%	98.0%	89.7%
**Duplicate**	0.5%	0.6%	0.6%	0.6%
**Fragment**	0.5%	0.5%	0.4%	7.3%
**Missing**	1%	1%	1%	2.5%
***n***	3354	3354	3354	3354
**Sauropsida**				
**Complete**	97.2%	97.2%	97.1%	92.6%
**Single**	96.4%	96.4%	96.4%	91.9%
**Duplicate**	0.8%	0.7%	0.7%	0.7%
**Fragment**	0.3%	0.3%	0.3%	3.3%
**Missing**	2.5%	2.5%	2.6%	4.1%
***n***	7480	7480	7480	7480

The statistics for the latest NCBI RefSeq version of the *P. bivittatus* genome are provided for comparison. Both the vertebrata_odb10 and sauropsida_odb10 databases were used for BUSCO analysis. *n* represents the number of BUSCOs in the database searched. The % of BUSCOs from each database identified in the genome as complete (single-copy and duplicated/multiple-copy), fragmented, or missing from the genome are indicated.

### Repeat annotation

**Fig. 3.**
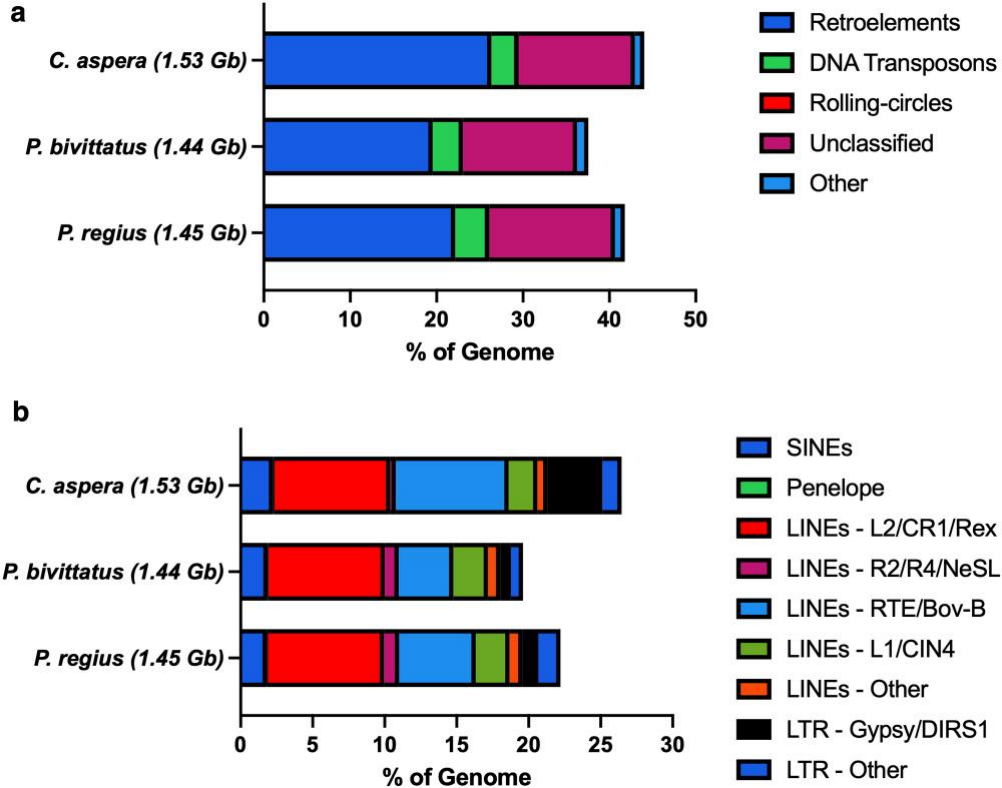
Repetitive DNA content of the *P. regius* genome. Total a) and retroelement b) repetitive DNA content of the *P. regius* genome with *P. bivittatus* and *C. aspera* for comparison. Genome sizes are indicated in parentheses. Repeat classes represent those identified by RepeatMasker. The database used for masking was a combination of the de novo *P. regius* library from RepeatModeler and repeats belonging to serpentes and serpentes descendants from the Dfam database.

The repeat element content of snake genomes can vary significantly, with reported ranges from 25% to 73% (Ahmad et al. 2021). To characterize the repeat landscape of the *P. regius* genome assembly, we constructed a de novo *P. regius* repeat library using RepeatModeler. This library contained 1,888 sequences with a minimum length of 32 bp, a maximum length of 13,774 bp, and an average length of 734.5 bp. We combined this library with repeats belonging to the serpentes taxon and all subtaxons from the Dfam database. The final library contained 45,117 sequences with a minimum length of 31 bp, a maximum length of 48,723 bp, and an average length of 1,377.4 bp. We masked 41.80% of the final *P. regius* assembly, similar to the 44.1% for *C. aspera* and slightly higher than the 37.6% calculated for *P. bivittatus* (Table 3; Fig. 3a). It should be noted that this value for *P. bivittatus* is higher than the 31.8% previously reported (Castoe et al. 2013). This is likely a consequence of using different libraries for repeat annotation, which underscores the importance of using the same library when comparing repeat content between species. The majority of *P. regius* repeats were retroelements (22.15%), followed by unclassified elements (14.52%), and DNA transposons (3.93%). Recent evidence suggests a correlation between genome size and repeat content in snakes, primarily driven by L2 and CR1 repetitive sequences (Peng et al. 2023). Consistent with this, L2/CR1/REX repeat content (8.04% to 8.07%) and genome size (1.44 to 1.53 Gb) are remarkably similar between the 2 Pythonidae species and *C. aspera*, a member of the Boidae family (Fig. 3b). These values are on the lower end when compared to other snake species (Castoe et al. 2011; Peng et al. 2023). The major driver of increased repeat content in ball pythons compared to Burmese pythons appears to be a larger presence of RTE-BovB elements (Fig. 3b). Along with Gypsy/DIRS1 LTR elements, these elements also account for the increased repeat content in *C. aspera* compared to *P. regius*.

**Table 3.** Repetitive DNA content of the *P. regius* genome.

Name	Number of elements in genome	Cumulative length (bp)	% of genome
**Retroelements**	1,231,220	320,776,211	22.15
SINEs	200,568	26,491,207	1.83
Penelope	7,555	845,127	0.06
LINEs	915,421	255,986,408	17.68
CRE/SLACS	0	0	0
L2/CR1/Rex	526,971	116,888,035	8.07
R1/LOA/Jockey	219	154,690	0.01
R2/R4/NeSL	27,888	15,018,164	1.04
RTE/BovB	205,367	77,122,561	5.33
L1/CIN4	84,349	34,044,971	2.35
LTR elements	115,231	38,298,596	2.64
BEL/Pao	1,590	175,117	0.01
Ty1/Copia	3,339	3,115,926	0.22
Gypsy/DIRS1	44,372	16,001,891	1.11
Retroviral	26,000	7,077,524	0.49
DNA transposons	310,426	56,971,884	3.93
hobo-Activator	139,870	14,617,838	1.01
Tc1-IS630-Pogo	160,739	40,504,151	2.8
En-Spm	0	0	0
MULE-MuDR	396	23,015	0
PiggyBac	169	12,755	0
Tourist/Harbinger	1,333	79,388	0.01
Other (Mirage, P-element)	345	29,029	0
Rolling-circles	6,590	751,413	0.05
Unclassified	1,560,011	210,265,874	14.52
**Total interspersed repeats in genome**		**588,859,096**	**40**.**66**
Small RNA	13,498	1,512,723	0.10
Satellites	1,694	209,365	0.01
Simple repeats	326,024	13,430,161	0.93
Low complexity	29,640	1,364,915	0.09
**Total repetitive DNA masked in genome**		**605,309,865**	**41**.**80**

The bolded values represent cumulative values.

### Genome annotation and orthologous cluster analysis

RNA from 9 different tissues was extracted and sequenced via Illumina and Nanopore cDNA sequencing. Across the 9 tissues, we generated 37,164,799 Nanopore reads with an average read length N50 of 705.2 bp, as well as over 592 million high-quality Illumina read pairs (see Supplementary Tables 3 and 4 in Supplementary File 1). We combined ab initio gene predictions, homology-based gene prediction, protein alignments, and RNA-seq-derived transcript alignments to generate a final genome annotation (Supplementary File 10). The final annotation was filtered to remove incomplete gene-coding models (partial models) and any transcripts that did not receive a functional annotation from EnTAP (either by similarity searching or gene family/ontology analysis). We identified 21,819 protein-coding genes with 44,849 mRNA transcripts, 37,900 unique coding sequences, and 37,856 unique protein products (Table 4; see Supplementary Files 11 and 12 for coding and protein sequences). We utilized tRNAscan-SE and Infernal cmscan to predict tRNAs and ncRNAs, respectively. The final annotation contains a total of 291 tRNAs and 1,111 ncRNAs (see Supplementary Figs. 1 and 2 in Supplementary File 1). This included 557 small nuclear RNAs (snRNA), 175 small nucleolar RNAs (snoRNA), 137 precursor microRNAs (pre-miRNA), 77 miRNAs, and 14 long noncoding RNAs (lncRNA).

**Table 4.** Quantitative summary of the final *P. regius* genome annotation.

Feature	*P. regius*	*P. bivittatus*	*C. aspera*
Protein-coding genes	21,819	19,793	18,397
mRNA transcripts	44,849	32,724	29,719
Unique CDSs	37,900	28,065	27,273
Unique proteins	37,856	27,951	27,137
Mean exons per mRNA	10.2	11.1	12.0
Mean exons per CDS	9.2	10.5	11.5
Mean introns per mRNA	9.2	10.1	11.0
Mean introns per CDS	8.2	9.5	10.5
Mean gene length (bp)	35,056	28,391	40,478
Median gene length (bp)	15,148	16,130	18,209
Mean mRNA length (bp)	40,047	33,469	46,392
Median mRNA length (bp)	18,712	19,728	23,254
Mean CDS length (bp)	1,633	1,765	1,909
Median CDS length (bp)	1,152	1,302	1,440
Mean exon length (bp)	348	276	260
Median exon length (bp)	133	131	132
Mean intron length (bp)	3,954	3,008	3,953
Median intron length (bp)	1,334	1,283	1,332

The number of protein-coding genes is higher than that of *P. bivittatus* (19,793) and *C. aspera* (18,397) but lies within the range from a recent study of 14 de novo snake genome assemblies by Peng et al. (18,301 to 23,299 protein-coding genes). The number of mRNA and unique coding sequences greatly exceeds that for the *P. bivittatus* RefSeq genome. The increase in total mRNA transcripts can partly be attributed to transcripts with different UTRs. We also identified approximately 10,000 more CDSs than in the existing *P. bivittatus* annotation, mostly due to an increased number of isoforms per gene in our annotation. It is likely that some of these transcripts are not expressed at physiologically relevant levels. However, because we did not have RNA-sequencing data from all possible tissue types, different developmental/maturity stages, or other biological conditions, filtering out transcripts based on expression levels may potentially throw out “real” transcripts that are more abundant in different biological contexts. Mean/median exon/intron counts per feature, as well as feature lengths, were largely comparable between the 2 python species and the viper boa (Table 4).

**Fig. 4.**
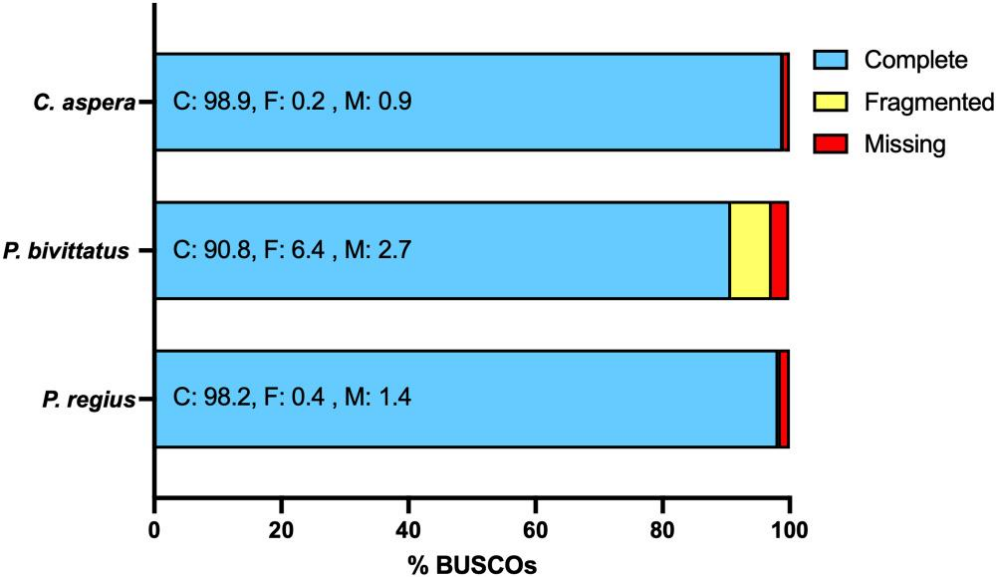
Proteome BUSCO scores for *P. regius*, *P. bivittatus*, and *C. aspera*. BUSCO scores were calculated using the vertebrata_odb10 dataset. Numbers in the bar plot represent percentages of BUSCOs identified as complete, fragmented, or missing. The numbers of single/duplicate complete BUSCOs are not provided, because isoforms lead to inflated duplicate percentages and taking only the longest isoforms leads to decreased overall complete BUSCOs since the longest isoform is not always the best isoform.

**Fig. 5.**
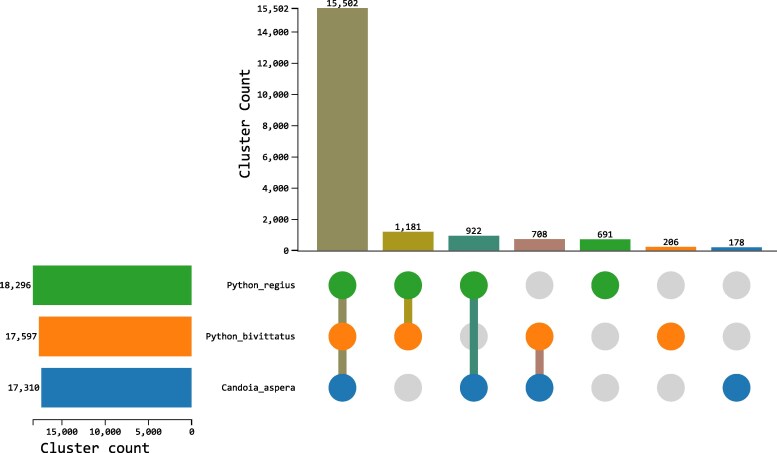
Orthologous cluster analysis of *P. regius*, *P. bivittatus*, and *C. aspera* proteomes. Upset plot indicates the number of clusters unique to each species or shared between species.

BUSCO analysis of the *P. regius* proteome yielded a complete score of 98.2%, significantly higher than the 90.8% for the *P. bivittatus* RefSeq set of proteins and comparable to the 98.9% complete score for the high-quality *C. aspera* RefSeq annotation (Fig. 4). Consequently, our annotation is more complete than the Burmese python RefSeq annotation and contains complete protein sequences that have not previously been annotated in a Pythonidae species. We identified 19,388 orthologous clusters, 15,502 (80.0%) of which were shared between the 3 species (Fig. 5). One thousand one hundred eighty-one clusters were specific to the 2 python species, 691 unique to *P. regius*, and 206 unique to *P. bivittatus*. The clusters unique to 1 of the 2 python species likely represent a combination of unannotated proteins in the other species, novel isoforms, and potentially novel species-specific genes that may contribute to species-specific biology.

## Conclusion

We present the first genome assembly for the ball python, representing one of the highest quality genome assemblies to date for a member of the Pythonidae family. Furthermore, until now, the Burmese python is the only member of this family to have had its genome annotated. Reflected by BUSCO analysis of the proteome, we present the most complete annotation of protein-coding genes for a python species. From a general standpoint, this work lays the foundation for further exploration of genetic diversity among snakes and broader evolutionary questions in the field of developmental biology. It will also aid in studies seeking to identify the molecular basis behind resistance to muscular atrophy, as well as the ability of pythons to undergo significant, rapid, and reversible cardiac remodeling following a meal. Such studies may provide unique insights into mechanisms of muscular atrophy and heart disease in humans and open potential avenues for novel therapeutics targeted toward these conditions.

## Data Availability

The raw sequencing data and BioSample information are available at NCBI under BioProject ID PRJNA1217506. This Whole Genome Shotgun project has been deposited at DDBJ/ENA/GenBank under the accession JBNNPY000000000 (pending) and is also available in the Supplementary material. Supplementary File 1 contains the supplementary figures and tables. Supplementary Files 2 and 3 contain the RepeatModeler de novo *P. regius* repeat library in FASTA and .stk format, respectively. Supplementary File 4 contains the RepeatMasker output. Supplementary File 5 contains the custom Python script to fix merged genes in the gff3 file. Supplementary File 6 contains the Python script to identify potential merged genes in the annotation based on genes containing CDSs that do not overlap at all. Supplementary File 7 contains the Python script to remove redundant mRNAs that do not have UTRs. Supplementary File 8 is the final mitogenome identified by MitoHiFi in GenBank format. Supplementary File 9 contains the assembly scaffold sequences in FASTA format. Supplementary File 10 is the final annotation in GFF3 format. Supplementary Files 11 and 12 contain the CDS and protein sequences extracted from the annotation, respectively. Previously published RNA-seq data used for annotation (Crocini et al. 2024) are available through the Gene Expression Omnibus under the accession code GSE273554. Supplementary material available at Figshare: https://doi.org/10.25387/g3.29874290.
